# Digital solutions in acute medicine: Will electronic health records join up the patient journey (soon?)

**DOI:** 10.1016/j.fhj.2026.100538

**Published:** 2026-06-25

**Authors:** Christian P. Subbe, Anne Kinderlerer, Yogini H. Jani

**Affiliations:** a Department of Advanced Internal Medicine, Woodlands Hospital, Singapore; b North Wales Medical School, Bangor, UK; c Chair of Unplanned Care, Kingston Hospital NHS Foundation Trust, Kingston upon Thames, UK; d Royal College of Physicians, London, UK; e Centre for Medicines Optimisation Research and Education, University College London Hospitals NHS Foundation Trust, London, UK; f Research Department of Practice and Policy, University College London School of Pharmacy, London, UK; g NIHR Central London Patient Safety Research Collaboration, University College London Hospitals NHS Foundation Trust, London, UK

**Keywords:** Electronic health records, Acute medicine, Usability, Improvement science, Patient safety, Burnout

## Abstract

Acute medicine provides care to patients during the first 72 h of their hospital stay. Clinicians face unique challenges of complexity in having to care simultaneously for groups of patients with multiple medical conditions and often complex needs. Digital innovation is central to modern clinical practice, yet its capacity to improve quality of care remains contested. This narrative review explores what acute physicians require from electronic health record (EHR) systems, drawing on evidence from usability studies, implementation evaluations and quality frameworks. Despite substantial investment, evidence of benefits for clinical outcomes, cost-effectiveness and user satisfaction remains modest. There is limited research on impact on mortality, efficiency and patient experience. Most research demonstrates inconsistent effects on process and intermediate outcomes such as clinical documentation quality (legibility) or improvements in medication safety. Poor system design based on work as described/imagined, rather than work as actually done, contributes to clinician burnout and undermines system adoption. Despite this, the review identifies good practice examples that show the potential for transformation in this challenging area of medical care. Future progress depends on integrating human factors science, rigorous usability assessment and responsive design. Research will need to evidence equity and sustainability. Acute physicians should ‘expect more’: systems that provide the right information, in the right context, at the right time – first time.

## Highlights

• EHRs offer benefits for medication safety in acute medicine but inconsistent evidence for overall quality improvement.
• Poor usability contributes significantly to clinician burnout and inefficiency in acute care.
• Rigorous usability testing at the hospital front door and improvement science should guide the next generation of EHR design.

## Introduction

### Digital solutions are key enablers of acute medical care

Iterative, organic developments over the last 25 years have led to multidimensional digital health records, from simple electronic documentation to more integrated and increasingly artificial intelligence (AI)-driven functionality, such as requesting tests, reporting diagnostics, prescribing and targeted prioritisation of at-risk patients, eg, those with acute kidney injury.^1^ The future is exciting as we get more joined-up digital systems, providing the potential for sharing records locally, nationally and internationally.

### The case for digital solutions in acute medicine

The goal of acute medicine is ‘better care of patients who present with acute medical illnesses’.^2^ ‘Better’ can be described through the six domains of quality as safe, effective, patient centred, timely, efficient and equitable.^3^ Acute medicine focuses on care in the first 72 h of a medical emergency admission to hospital. Practically, this requires accurate information for diagnosis, management, appropriate monitoring plans, handover and follow-up between teams of healthcare professionals and the patient.

Digital innovation is seen as a key solution in constrained healthcare systems. Digital solutions are multi-faceted, with an increasing breadth of use cases, such as electronic health records (EHRs) accessible using smartphone applications, handheld ultrasound, telemedicine and wearable monitoring devices in ambulatory care. In this manuscript we focus on EHRs as a pivotal requirement to connect the patient journey in multidisciplinary and multi-provider health ecosystems.

Medical documentation has long been integral to clinical care. Initial standardisation of medical records was advocated by Lloyd George for insurance purposes as much as clinical documentation. Functional ‘ideal’ records are expected to be accurate, concise and readily accessible. Contemporary expectations have expanded to include interoperability, decision support and contribution to research.

In the UK, national initiatives demonstrated how ambition can exceed delivery.^4^, ^5^ Despite investments exceeding £12 billion the aspiration of a national digital health record was not achieved. The implementation of digital records has been heterogeneous and digital maturity across NHS trusts remains highly variable.^6^

This narrative review explores what clinicians working in acute medicine may require from EHR systems to deliver high-quality healthcare and compares it to available evidence.

## Evidence base for digital solutions in acute medicine

Research on EHRs in acute medicine falls broadly into two groups: usability studies that are often undertaken in lab environments, and studies that examine impact of implementation in clinical care.

### Usability

Usability determines safety, system performance, and user satisfaction. Studies with nurses and doctors working in acute care show that usability of EHRs can be hindered by poor formatting of screens and information overload, the latter made worse by duplication and redundancy of information.^7^

Several frameworks provide structure for assessing EHR usability assessment during procurement and testing, but their adoption is limited.^8^ The NASA Task Load Index, a widely used, validated tool to measure workload, can be used to demonstrate correlation between usability, cognitive load and error rates.^9^ Comparative studies reveal up to ninefold differences in task completion time between commercial EHRs.^10^

Importantly, user experience is directly linked to safety. In a study across 12 US hospitals, higher user experience scores correlated with better health information technology safety ratings.^11^ However, evaluation of user experience scores and usability measures during implementation remains heterogeneous, with more than 70 different usability metrics reported in research.^12^

### Effects of implementation on domains of quality

There is variable evidence of the effect of EHRs across the different domains of quality,^3^ and some unintended consequences, such as new types of harms or errors and clinician burnout, have emerged.

Efficiency of EHRs remains debated: while there is evidence of improvement in documentation completeness,^13^ documentation time for doctors and other healthcare professionals may increase despite theoretical savings,^14^, ^15^, ^16^, ^17^ leaving clinicians with ‘4,000-click’ shifts.^18^ Timeliness improves for some processes, but prescribing delays remain problematic.^11^

Evidence for effectiveness and impact of implementation on mortality is inconsistent and contextual; hospitals that adopt EHRs early may already be high performing.^19^ In contrast, a US paediatric ICU reported increased mortality immediately following EHR implementation.^20^

Limited research is available on cost-effectiveness in acute care: while systematic reviews show reduced test ordering and shorter stays in the emergency department, these are contrasted with higher admission rates.^21^ In a single study published by an EHR provider, shorter length of stay was found for patients who use EHR-associated patient portals, but findings were not adjusted for patient factors such as social deprivation or digital literacy, or for differences between hospitals.^22^

Safety improvements are well documented in medication management: systematic reviews show reductions in prescribing errors and rates of adverse events,^23^ but EHR-specific harms such as alert fatigue and communication failure are emerging.^24^ Clinician burnout has become a major unintended consequence of digitisation. A systematic review of 29 studies found that poor usability, unreliable performance and inflexible workflows contributed to stress and disengagement of clinicians, and emergency physicians are particularly affected.^25^

Patient-centred outcomes are largely unchanged,^26^ and equity effects remain unexplored. Sustainability gains from reduced paper use may be more than offset by server energy demands.^27^

## What should the future focus be?

### Unique challenges in acute medical care

Acute medicine as a specialty faces a fairly unique challenge: there is an expectation that an acute medicine team may look after up to 60 acutely unwell patients at the same time, with real-time changes in severity of illness in multiple patients. Most of these have interfaced with a number of specialties or healthcare organisations. EHRs need to be able to assist clinicians in guiding workflow across multiple patients and reducing rather than increasing cognitive load. Given that a small number of clinical conditions make up the bulk of scenarios that are managed, digital solutions provide opportunities for streamlining care delivery, facilitated by basic or advanced clinical decision support and establishing standard care pathways.^28^ Examples are summarised in Table 1 (below).

**Table 1:** Case examples of digital systems in acute medical care.

Theme	Example	Potential benefits	Gaps in evidence/challenges
**Transmission of information across interfaces**	Triage systems at the front door of the hospital that collect NEWS score, frailty score, 4AT etc.	Potential to direct patients directly to appropriate services including frailty experts.^29^	Lacking data on feasibility and effects on frailty pathways from triage. Missing of documentation on functional status and social support in current systems might limit impact.
	Digital lists of patients and handover actions	Suggested improvement in flow and quality indicators.^30^	Reliability of process, dependence on local context unclear.
	Regional shared care records	Visibility of patient data from primary care and other secondary care providers for clinicians resulting in reduced time for documentation and financial savings^31^	Compatibility between systems from different providers. Missing evidence for measured impact in clinical outcomes and efficiencies.
**Alternative documentation systems**	Electronic clerking proformas	Compliance with documentation standards improved.^32^	Little work has occurred in digital systems to describe such tools or to standardise them across organisations or systems
	Ward round checklist	More reliable documentation.^33^	Little evidence on how to best implement in electronic systems
	Ambient voice technology for documentation.	Increased productivity and more time for direct patient care^34^	Evidence on how to use this to document ward practice is lacking.
**Detection of risk**	Standardised systems to detect risk from sepsis	Reduced mortality^35^	Best way to display data and how and to whom to alert is unproven.
**Interpretation of pathology results**	Review of abnormal liver functions test by an AI system with automated booking of further tests and referrals.	Increased efficiency and reliability of process^36^	Capacity across the whole pathway.

The interface between community and hospital is possibly one of the most dynamic areas in healthcare linking multiple providers, professions and specialties with continuously evolving standards of practice. Adapting electronic platforms to latest standards of care at pace is often technically possible, but requires significant resources (time and cost). Rapid plan-do-study-act cycles that are the NHS’s ‘industry standard’ for improvement are therefore challenging. Clinicians often struggle to adapt EHRs as transformative tools for clinical work and safer, more effective and humane care.

### Human(e) interfaces

Acute physicians seek systems that enhance quality – accurate, timely information that supports diagnosis, communication and decision-making. Yet many systems add cognitive and administrative burden, leading to frustration and inefficiency.^18^ Addressing burnout might require redesigning systems using a human factor engineering approach, considering clinical workflow and work as done, rather than administrative or reporting needs alone. There are a number of gaps in the available evidence; in particular, current solutions might not focus enough on the computer–human interface.

New frameworks for evaluation are emerging that centrally feature evidence, usability, and privacy and security.^37^ Other important evidence gaps are in areas of equity and sustainability: in terms of development as well as clinical impact.

### Regulatory boundaries

Current systems are bound by existing regulations and legislation, which vary across countries. Most EHRs are commercial products, provided by vendors in one country, typically the USA and used across the world. Functions that may shift the workload in acute medicine, such as telemedicine, AI and clinical decision support, cross multiple regulatory domains including medical device standards and information governance legislation. Patients’ engagement, consent and use of digital solutions such as patient portals or wearables for monitoring and managing their own care also require further work. Too often their access is limited by technology or jargon.

The public remains sceptical and there are concerns how the implementation of AI solutions will change the relationship of trust between patients and healthcare professionals.^38^ Legal frameworks to introduce AI need to be transparent to be trustworthy and link impact to a proportionate risk of implementation.^39^

### Science driven development

EHR development must evolve from static systems to adaptive, learning platforms. Techniques such as A/B testing – already used in web design – can evaluate small interface changes in real time.^40^ Behavioural ‘nudge’ approaches show promise for improving test and medication ordering, though long-term impact is uncertain.^41^ AI tools for alerts and event detection remain in early stages, with few high-quality trials.

## Moving forward

**Fig. 1:**
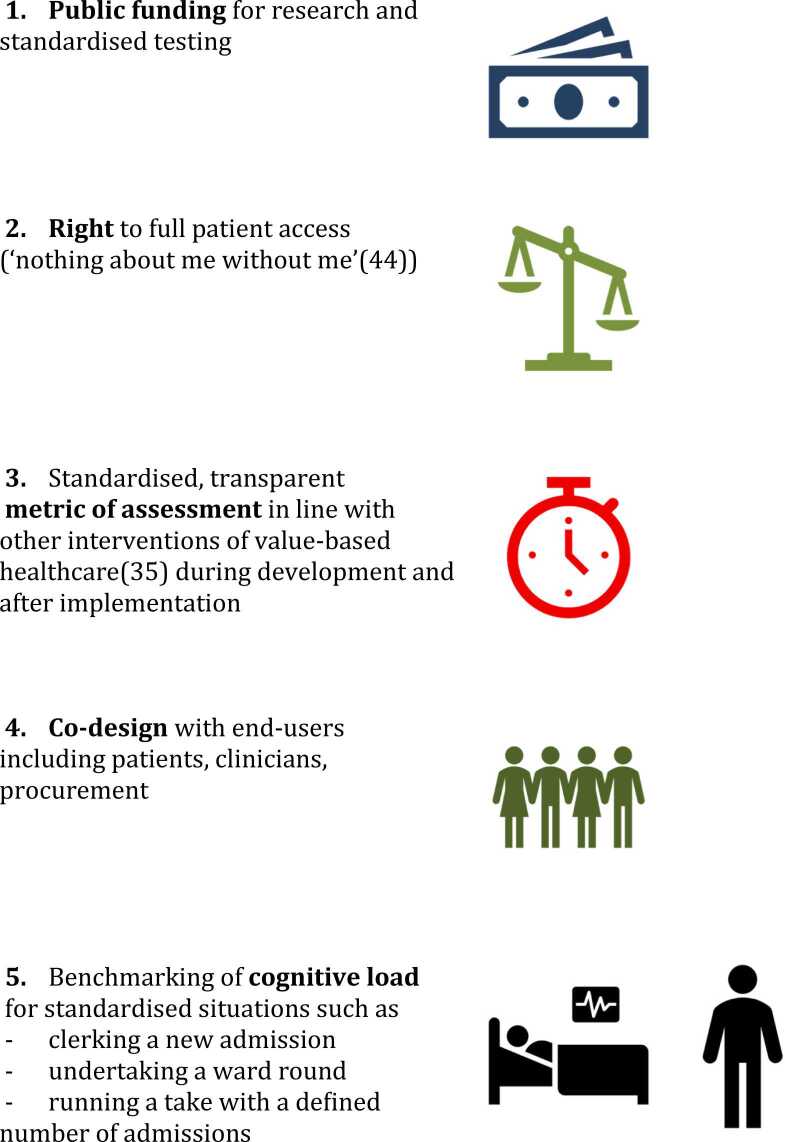
Five steps towards digital solutions for high-quality healthcare.

Clinicians in acute medicine want electronic notes that enhance quality of care for patients, staff and healthcare systems. We hypothesise that strengthening of structures and processes in the development of EHRs would be helpful to improve outcomes for high-quality healthcare^42^ at the hospital front door and beyond (Fig. 1):

1. **Provide public funding** for research and evaluation**:** EHRs are among the costliest investments for healthcare organisations. In line with other areas of innovation, funding for procurement needs to be matched with funding for research to assure that public spend is value for taxpayers and that systems are appropriate for the UK healthcare setting. This research needs to explicitly include areas with the highest work pressures and burnout, such as intensive care and acute medical care.
2. **Strengthen right of access for patients:** while many EHRs already provide access for patients, utility might be limited by digital literacy. Patient empowerment and engagement in their own care are especially important, with increasing funding shortages in healthcare and the increasing complexity of patients seen in acute care. This needs to be reflected in the way that EHRs are designed and made accessible.
3. **Standardise metric of assessment** for EHRs in line with other interventions of value-based healthcare^43^: while quality and safety feature highly in advertising of EHRs, metrics for measuring value need to be standardised, patient centric and reproducible. In line with the development of pharmaceuticals, post-implementation surveillance of products is crucial.
4. **Co-design** with end users needs to be front and centre of development. Design needs to stay in step with the pace of dynamic changes in acute care. Current systems often struggle to ingest information from different sources and present it meaningfully. What is often missing is a visual interface that is appropriate for clinicians in high-pressure environments, looking after multiple patients or, indeed, patients with limited health or digital literacy.
5. **Benchmark metrics of cognitive load.** This is a key metric for patient safety (and staff burnout). While synthesising information from multiple specialties and systems, acute medicine is particularly challenging in this area but has standardised building blocks such the admission of an individual patient, the post-take ward round, or the running of the ‘medical take’ for a whole hospital. Despite the differences in hospitals, these core features are often surprisingly similar even internationally. Benchmarking cognitive load in these settings may therefore help with design refinements.

Design and improvement science provide frameworks for defining aims, measuring change and continuous learning. Application of these frameworks, which include guidance on co-design with multiple stakeholders and methods for rapid iterative development of prototypes, is essential to build systems that improve outcomes and efficiency, while assuring staff wellbeing and safety of data.

It is time for policy makers to assure that regulators, funders and professional societies collaborate closer with users for EHRs to deliver on their promise of a ‘better’, safer, joined-up patient journey, especially in the dynamic high-turnover area of acute medical care.

## CRediT authorship contribution statement

**Christian P. Subbe:** Writing – review & editing, Writing – original draft, Visualization, Methodology, Conceptualization. **Anne Kinderlerer:** Writing – review & editing. **Yogini H. Jani:** Writing – review & editing, Writing – original draft, Methodology, Conceptualization.

## Funding

This research did not receive any specific grant from funding agencies in the public, commercial, or not-for-profit sectors.

## Declaration of generative AI and AI-assisted technologies in the writing process

During the preparation of this work, the authors used ChatGPT in order to summarise a PowerPoint presentation that CP Subbe had done on the topic of this article. After using this tool, the authors reviewed and edited the content and take full responsibility for the content of the published article.

## Declaration of competing interest

The authors declare the following financial interests/personal relationships which may be considered as potential competing interests: Anne Kinderlerer is Royal College of Physicians clinical digital lead, London, UK. If there are other authors, they declare that they have no known competing financial interests or personal relationships that could have appeared to influence the work reported in this paper.

## References

[1] Chew C.K.T., Hogan H., Jani Y. (2021). Scoping review exploring the impact of digital systems on processes and outcomes in the care management of acute kidney injury and progress towards establishing learning healthcare systems. BMJ Health Care Inform.

[2] Society for Acute Medicine Objectives [Internet]. [cited 2026 Jan 9]. Available from: 〈https://www.acutemedicine.org.uk/sam-membership/〉.

[3] Institute of Medicine (U.S.) (2001). Crossing the Quality Chasm.

[4] Robertson A., Bates D.W., Sheikh A. (2011). The rise and fall of England’s national programme for IT. J R Soc Med.

[5] Coiera E.W. (2007). Lessons from the NHS national programme for IT. Med J Aust.

[6] Digital Maturity Assessment report 2024 and 2025 results - NHS England Digital [Internet]. [cited 2026 Mar 29]. Available from: 〈https://digital.nhs.uk/data-and-information/digital-maturity-assessment-report−2024-and-2025-results〉.

[7] Drews S., Thompson C., Gerlach H. (2025). Usability of electronic health records by clinicians in acute care: a scoping review. Can J Nurs Inform.

[8] Tyllinen M., Kaipio J., Lääveri T. (2018). Studies in Health Technology and Informatics.

[9] Mazur L.M., Mosaly P.R., Moore C., Marks L. (2019). Association of the usability of electronic health records with cognitive workload and performance levels among physicians. JAMA Netw Open.

[10] Ratwani R.M., Zachary Hettinger A., Kosydar A., Fairbanks R.J., Hodgkins M.L. (2017). A framework for evaluating electronic health record vendor user-centered design and usability testing processes. J Am Med Inform Assoc.

[11] Classen D.C., Longhurst C.A., Davis T., Milstein J.A., Bates D.W. (2023). Inpatient EHR user experience and hospital EHR safety performance. JAMA Netw Open.

[12] Wronikowska M.W., Malycha J., Morgan L.J. (2021). Systematic review of applied usability metrics within usability evaluation methods for hospital electronic healthcare record systems. J Eval Clin Pract.

[13] Wurster F., Fütterer G., Beckmann M. (2022). The analyzation of change in documentation due to the introduction of electronic patient records in hospitals—a systematic review. J Med Syst.

[14] Poissant L., Pereira J., Tamblyn R., Kawasumi Y. (2005). The impact of electronic health records on time efficiency of physicians and nurses: a systematic review. J Am Med Inform Assoc.

[15] Bakhoum N., Gerhart C., Schremp E. (2021). A time and motion analysis of nursing workload and electronic health record use in the emergency department. J Emerg Nurs.

[16] McLeod M., Karampatakis G.D., Heyligen L., McGinley A., Franklin B.D. (2019). The impact of implementing a hospital electronic prescribing and administration system on clinical pharmacists’ activities - a mixed methods study. BMC Health Serv Res.

[17] Harmon C.S., Adams S.A., Davis J.E., Donevant S.B., Gephart S.M. (2025). Psychometric evaluation of cognitive load and unintended consequences of electronic health records in US emergency nurses. Comput Inform Nurs.

[18] Hill R.G., Sears L.M., Melanson S.W. (2013). 4000 Clicks: a productivity analysis of electronic medical records in a community hospital ED. Am J Emerg Med.

[19] Lin S.C., Jha A.K., Adler-Milstein J. (2018). Electronic health records associated with lower hospital mortality after systems have time to mature. Health Aff.

[20] Han Y.Y., Carcillo J.A., Venkataraman S.T. (2005). Unexpected increased mortality after implementation of a commercially sold computerized physician order entry system. Pediatrics.

[21] Mullins A., O’Donnell R., Mousa M. (2020). Health outcomes and healthcare efficiencies associated with the use of electronic health records in hospital emergency departments: a systematic review. J Med Syst.

[22] Rasmussen D., Gallagher K., Goldsmith A., Lindemann P., Barkley E. (2021). Shorter hospital stays associated with patient portal use. Epic Res.

[23] Campanella P., Lovato E., Marone C. (2016). The impact of electronic health records on healthcare quality: a systematic review and meta-analysis. Eur J Public Health.

[24] Subbe C.P., Tellier G., Barach P. (2021). Impact of electronic health records on predefined safety outcomes in patients admitted to hospital: a scoping review. BMJ Open.

[25] Alobayli F., O’Connor S., Holloway A., Cresswell K. (2023). Electronic health record stress and burnout among clinicians in hospital settings: a systematic review. Digit Health.

[26] Jarvis B., Johnson T., Butler P. (2013). Assessing the impact of electronic health records as an enabler of hospital quality and patient satisfaction. Acad Med.

[27] Kwon C., Essayei L., Spencer M. (2024). The environmental impacts of electronic medical records versus paper records at a large eye hospital in India: life cycle assessment study. J Med Internet Res.

[28] Subbe C.P., Bottle R.A., Bell D. (2011). Acute medicine: triage, timing and teaching in the context of medical emergency admissions. Eur J Intern Med.

[29] Craggs H. (2022). 1 Digital transformation of the acute medical take – improving standards of care. BMJ Health Care Inform.

[30] OneLondon reports savings from London Care Record | UK Authority [Internet]. [cited 2026 Jan 15]. Available from: 〈https://www.ukauthority.com/articles/onelondon-reports-savings-from-london-care-record〉.

[31] Mohideen A.T., Foxall-Smith M., Arnaouti M. (2025). A standardised clerking proforma improves the perceived satisfaction of healthcare professionals and leads to better documentation. Cureus.

[32] Hale G., McNab D. (2015). Developing a ward round checklist to improve patient safety. BMJ Qual Improv Rep.

[33] Ambient Voice Technology. AI-Scribe’ FAQs | Great Ormond Street Hospital [Internet]. [cited 2026 Jan 15]. 2023;30(7):1313-1322. Available from: 〈https://www.gosh.nhs.uk/about-us/our-strategy/our-ai-strategy-for−2025-2028/ambient-voice-technology-ai-scribe-faqs/〉.

[34] Honeyford K., Nwosu A.P., Lazzarino R. (2023). Prevalence of electronic screening for sepsis in National Health Service acute hospitals in England. BMJ Health Care Inform.

[35] Nobes J., Leith D., Handjiev S., Dillon J.F., Dow E. (2024). Intelligent liver function testing (iLFT): an intelligent laboratory approach to identifying chronic liver disease. Diagnostics.

[36] Zelmer J. (2019). Nothing about me without me. Healthc Policy.

[37] Goldsack J.C., Holliday C., Sharma Y., Mirsky D., Zanetti C., Vandendriessche B. (2025). Development of an evidence-based evaluation framework for digital health software products. Sci Rep.

[38] AI in health care: what do the public and NHS staff think? - The Health Foundation [Internet]. [cited 2026 Jan 15]. 2025;17(12):e98706. Available from: 〈https://www.health.org.uk/reports-and-analysis/analysis/ai-in-health-care-what-do-the-public-and-nhs-staff-think〉.

[39] Professor Alastair Denniston: the future regulation of AI in healthcare - GOV.UK [Internet]. [cited 2026 Jan 15]. Available from: 〈https://www.gov.uk/government/news/professor-alastair-denniston-the-future-regulation-of-ai-in-healthcare〉.

[40] Austrian J., Mendoza F., Szerencsy A. (2021). Applying A/B testing to clinical decision support: rapid randomized controlled trials. J Med Internet Res.

[41] Raban M.Z., Gates P.J., Gamboa S., Gonzalez G., Westbrook J.I. (2023). Effectiveness of non-interruptive nudge interventions in electronic health records to improve the delivery of care in hospitals: a systematic review. J Am Med Inform Assoc.

[42] Adler-Milstein J., Embi P.J., Middleton B., Sarkar I.N., Smith J. (2017). Crossing the health IT chasm: considerations and policy recommendations to overcome current challenges andenable value-based care. J Am Med Inform Assoc.

[43] Moloney E., O’Donovan M.R., Burke D. (2025). Development of a novel frailty trigger for use at triage in the emergency department. Acad Emerg Med.

